# Accelerating cervical cancer elimination: healthcare decision-making and cervical cancer screening uptake in sub-Saharan Africa

**DOI:** 10.1016/j.pmedr.2026.103572

**Published:** 2026-07-15

**Authors:** Ololade Julius Baruwa, Mwiza Gideon Singini, Hlengiwe Gwebu, Chinelo Cynthia Nduka

**Affiliations:** a Department of Nursing and Public Health, University of Fort Hare, East London, South Africa; b Non-communicable Diseases Research Unit, South African Medical Research Council, Cape Town, South Africa; c Department of Community Medicine, Nnamdi Azikiwe University Teaching Hospital, Nnewi, Nigeria

**Keywords:** Cervical cancer, Screening, Healthcare decision-making, Women's autonomy, Sub-Saharan Africa

## Abstract

**Objectives:**

This study examined the association between healthcare decision-making and cervical cancer screening among ever-partnered women in SSA and assessed whether this relationship varies across countries.

**Methods:**

This cross-sectional study pooled Demographic and Health Survey data (2019–2024) from 114,764 ever-partnered women aged 25–49 years across fourteen SSA countries. Multivariable logistic regression models estimated adjusted odds ratios (aORs) and 95% confidence intervals (CIs). Country-by-healthcare decision-making interaction terms and adjusted predicted probabilities assessed contextual variation.

**Results:**

Overall, 13.00% of women reported ever being screened for cervical cancer. Compared with women making healthcare decisions independently, women whose partners made healthcare decisions alone (aOR = 0.85, 95% CI = 0.81, 0.91) and those involved in shared decision-making (aOR = 0.94, 95% CI = 0.90–0.99) had lower odds of screening. Screening uptake was higher among older, more educated, and wealthier women and those with media exposure, but lower among women with inequitable gender attitudes and living in rural areas and those reporting distance barriers. Significant country-level variation was observed.

**Conclusions:**

Healthcare decision-making is an important, context-dependent determinant of cervical cancer screening uptake in SSA. Context-specific, gender-responsive strategies that strengthen women's participation in healthcare decision-making are needed to accelerate cervical cancer elimination.

## Highlights

• Cervical cancer screening uptake remained low (13%) among ever-partnered women.
• Cervical cancer screening varied across the 14 sub-Saharan African countries.
• Healthcare decision-making is associated with cervical cancer screening uptake.
• Education, wealth, and media also increased cervical cancer screening uptake.

## 1 Introduction

Cervical cancer remains one of the most preventable yet deadly cancers among women globally, with a disproportionate burden borne by low- and middle-income countries (LMICs), particularly in sub-Saharan Africa (SSA) (Ferlay et al., 2024). According to GLOBOCAN 2022 estimates, approximately 660,000 new cases and 350,000 deaths were reported globally in 2022, with SSA contributing a substantial and disproportionate burden of both incidence and mortality due to persistent gaps in access to effective cancer prevention, early detection, and treatment services (Bray et al., 2024; World Health Organization, 2020). In response, the WHO launched the Global Strategy to Accelerate the Elimination of Cervical Cancer, calling for 70% of women to be screened with a high-performance test by the ages of 35 and 45 years (World Health Organization, 2020). However, progress toward this target has been uneven and slow across the region (Bruni et al., 2022).

Population-based evidence consistently shows that cervical cancer screening uptake in SSA remains critically low, often under 20% among eligible women, with marked disparities by age, education, socioeconomic status, residence, and access to health information (Ba et al., 2021; Asgedom et al., 2024; Baykemagn et al., 2025). Structural barriers such as limited screening infrastructure, shortages of trained personnel, weak referral systems, and geographic inaccessibility continue to constrain screening uptake (Mwaka et al., 2016; Petersen et al., 2022). Beyond these system-level constraints, increasing attention has been directed toward social and relational determinants, particularly gender norms and intra-household power dynamics, in shaping women's preventive healthcare utilisation (Heise et al., 2019; Chol et al., 2019).

This study is informed by Kabeer's empowerment framework and broader gender and power theories, which posit that access to healthcare is embedded within relational power structures and decision-making processes operating within households and communities (Connell, 2013; Kabeer, 1999). From this perspective, healthcare decision-making may represent an important mechanism through which gendered power relations influence women's access to preventive health services, including cervical cancer screening. In many SSA contexts, women's healthcare access is strongly influenced by entrenched gender inequalities and partner-only healthcare decision-making **(****Pratley, 2016****;** **Yaya et al., 2016****;** **Asaolu et al., 2018****)**. Greater decision-making autonomy has been associated with increased utilisation of maternal and reproductive health services, including antenatal care, facility delivery, and contraception (Pratley, 2016; Asaolu et al., 2018). However, evidence regarding healthcare decision-making and cervical cancer screening remains limited and inconsistent. While supportive partner engagement may facilitate healthcare access through financial or emotional support (Ditekemena et al., 2012; Tokhi et al., 2018; Moyo et al., 2024), situations in which women have limited or no involvement in healthcare decision-making may restrict their ability to independently seek preventive services (Baruwa et al., 2025; Negash et al., 2023; Ouahid et al., 2025).

Despite growing recognition of gendered power relations in health-seeking behaviour, most cervical cancer screening studies in SSA have focused primarily on individual socioeconomic or health-system factors, with relatively limited attention to healthcare decision-making dynamics (Ba et al., 2021; Baykemagn et al., 2025). Furthermore, many existing studies have been conducted in single-country settings, limiting understanding of how the association between healthcare decision-making and cervical cancer screening may vary across diverse sociocultural and health-system contexts (Pratley, 2016; Baruwa et al., 2025). Although a previous multicountry DHS study (Okyere et al., 2022) examined women's healthcare decision-making and cervical cancer screening in SSA, important gaps remain. First, the evidence predates several recent DHS surveys now available across the region. Second, little is known about whether the association between healthcare decision-making and cervical cancer varies across countries, despite substantial contextual differences in gender norms, health systems, and cervical cancer screening programmes. Understanding this contextual variation may provide important insights for designing context-specific cervical cancer prevention strategies. Using pooled Demographic and Health Survey (DHS) data from fourteen SSA countries collected between 2019 and 2024, this study examines whether partner-only healthcare decision-making is associated with cervical cancer screening uptake among ever-partnered women and whether this association varies across national contexts in SSA.

## 2 Methods

### 2.1 Study design and population

This cross-sectional study used pooled, nationally representative data from the DHS conducted between 2021 and 2024 in fourteen SSA countries: Burkina Faso (2021), Democratic Republic of Congo (2023–2024), Côte d'Ivoire (2021), Ghana (2022), Kenya (2022), Lesotho (2023–2024), Malawi (2024), Mali (2023–2024), Mauritania (2019–2021), Mozambique (2022−2023), Nigeria (2024), Senegal (2023), Tanzania (2022), and Zambia (2024). DHS surveys employ standardised questionnaires and field procedures across participating countries, enabling valid cross-country comparisons.

Data were obtained from the women's individual recode (IR) files, which contain information on women's sociodemographic characteristics, reproductive health indicators, and health service utilisation. DHS surveys employ a stratified two-stage cluster sampling design. Enumeration areas are first selected as primary sampling units using probability proportional to size, followed by systematic household selection within each cluster. Eligible women aged 15–49 years were interviewed using standardised survey instruments. The study used publicly available anonymised secondary data from the DHS Program. Ethical approval for each survey was obtained by the respective national ethics committees and the DHS Program Institutional Review Board before data collection. Permission to use the datasets was obtained from the DHS Program. Because the datasets are de-identified and publicly available, additional ethical approval for this secondary analysis was exempted in accordance with institutional guidelines. Detailed survey procedures are available in DHS methodological reports.

Consistent with World Health Organisation (WHO) recommendations prioritising cervical cancer screening among women aged 25–49 years (World Health Organization, 2025), the analysis was restricted to women within this age group. The study further focused on ever-partnered women because the main exposure variable relates specifically to partner involvement in healthcare decision-making. Women who were never married or never in union were excluded. The final analytic sample included 114,764 women with complete information on the outcome and key exposure variables.

### 2.2 Measures

The outcome was cervical cancer screening uptake, derived from the DHS question asking whether respondents had ever been tested for cervical cancer by a health professional. Responses were coded as 1 = Yes and 0 = No.

The main exposure was healthcare decision-making, assessed using the DHS item on who usually decides on the respondent's healthcare. Healthcare decision-making was classified into three mutually exclusive categories: (i) woman-only decision-making (independent healthcare decision-making) (ii) partner-only decision-making (no involvement by the woman), and (iii) shared decision-making (woman jointly with partner or another person).

Covariates were selected a priori based on literature linking women's socioeconomic characteristics, autonomy, and gender relations with sexual and reproductive healthcare utilisation in SSA (Ba et al., 2021; Asgedom et al., 2024; Baykemagn et al., 2025; Baruwa et al., 2025; Negash et al., 2023). These included *age* (25–29, 30–34, 35–39, 40–44, 45–49); *educational level* (none, primary, secondary or higher); *household wealth status*, measured using the DHS wealth index and categorised as poor, middle, and rich, and place of residence (urban vs. rural)*.* D*istance to a health facility* was measured based on the respondent's perception of whether distance to a health facility was a big problem or not, and media exposure was defined as access to at least one form of mass media (radio, newspaper, or television) and was coded as yes or no. Other variables include *early marriage* (before age 18), *adolescent motherhood* (childbirth before age 20)**,** and *inequitable gender attitudes* were measured using the DHS standard justification of wife-beating items, with women classified as holding inequitable attitudes if they endorsed at least one justification for a husband to beat his wife: (i) the wife goes out without telling the husband, (ii) she neglects the children, (iii) she argues with the husband, (iv) she refuses to have sex with him, or (v) she burns the food. *Country* was included as a fixed effect to account for contextual variation across settings.

### 2.3 Statistical analysis

Analyses were conducted in four stages. First, descriptive statistics were used to summarise respondent characteristics and the prevalence of cervical cancer screening. Second, a multivariable logistic regression model was fitted to examine the association between healthcare decision-making and cervical cancer screening uptake, with results reported as adjusted odds ratios (aORs) and 95% confidence intervals (CIs). Third, interaction terms between country and healthcare decision-making were included to assess whether the association varied across countries, and the overall interaction was evaluated using the Wald test (Supplementary Table S2). Lastly, predicted probabilities were estimated from the interaction model to facilitate interpretation of country-specific differences in cervical cancer screening uptake across healthcare decision-making categories. Prior to multivariate modelling, multicollinearity among the independent variables was assessed using the variance inflation factor (VIF). No evidence of problematic multicollinearity was observed (mean VIF = 1.30; supplementary Table S1). Statistical significance was set at *p* < 0.05. All statistical analyses were performed using Stata version 18.

## 3 Results

### 3.1 Characteristics of the study population

Overall, cervical cancer screening uptake was low, with only 13.00% of women reporting ever being screened, while 41.43% reported partner-only healthcare decision-making, 36.40% reported shared healthcare decision-making, and 22.18% reported making healthcare decisions independently. Women aged 25–29 years constituted the largest proportion of the sample (25.63%). About 37.77% of women had no formal education, and 42.98% belonged to rich households. Access to mass media was reported by 66.28% of women. Early marriage and adolescent motherhood remained common, affecting 39.20% and 49.10% of women, respectively. In addition, 34.41% justified wife-beating under at least one circumstance, reflecting persistent inequitable gender norms. Most respondents resided in rural areas (61.52%), while 34.17% reported distance to health facilities as a major barrier. Nigeria contributed the largest proportion of respondents (18.21%), followed by DR Congo (10.33%) and Burkina Faso (8.49%). (See Table 1).

**Table 1:** Characteristics of ever-partnered women aged 25–49 years in 14 sub-Saharan African countries using DHS data, 2019–2024.

Characteristic	n/N	Percentage
**Outcome variable**		
Cervical cancer screening	14,220/114,764	13.00
**Independent variables**		
**Healthcare decision-making**		
Woman-only decision-making	24,697/114,764	22.18
Partner-only decision-making	47,577/114,764	41.43
Shared decision-making	42,490/114,764	36.40
**Age group (years)**		
25–29	29,111/114,764	25.63
30–34	27,442/114,764	24.04
35–39	24,938/114,764	21.64
40–44	19,197/114,764	16.65
45–49	14,076/114,764	12.04
**Education**		
No education	44,866/114,764	37.77
Primary	32,835/114,764	28.53
Secondary/higher	37,063/114,764	33.70
**Wealth index**		
Poor	46,259/114,764	37.61
Middle	23,529/114,764	19.41
Rich	44,715/114,764	42.98

Media exposure	73,644/114,764	66.28
Early marriage	45,466/114,764	39.20
Adolescent motherhood	56,703/114,764	49.10
Inequitable gender attitudes	40,419/114,764	34.41
Rural residence	72,796/114,764	61.52
Distance problem to health facilities	41,315/114,764	34.17
**Country**		
Burkina Faso	9662/114,764	8.49
Democratic Republic of Congo	12,279/114,764	10.33
Côte d'Ivoire	7615/114,764	6.38
Ghana	7328/114,764	6.15
Kenya	7825/114,764	6.77
Lesotho	1261/114,764	1.14
Malawi	9133/114,764	7.83
Mali	9095/114,764	8.26
Mauritania	3618/114,764	3.22
Mozambique	5509/114,764	4.94
Nigeria	20,612/114,764	18.21
Senegal	8083/114,764	7.06
Tanzania	7064/114,764	6.19
Zambia	5680/114,764	5.03

n: sub-sample and N: total sample.

### 3.2 Multivariable analysis of factors associated with cervical cancer screening uptake

Results from the multivariable logistic regression analysis (Table 2) showed that healthcare decision-making was independently associated with cervical cancer screening uptake among ever-partnered women in SSA. Compared with women who made healthcare decisions independently, those whose healthcare decisions were made by their partners alone had 15% lower odds of cervical cancer screening (aOR = 0.85, 95% CI = 0.81, 0.91), while women involved in shared healthcare decision-making also had lower odds of screening (aOR = 0.94, 95% CI = 0.90, 0.99).

**Table 2:** Multivariable association between partner-only healthcare decision-making and cervical cancer screening uptake among ever-partnered women in 14 sub-Saharan African countries using DHS data, 2019–2024.

**Variable**	**aOR (95% CI)**
**Healthcare decision-making**	
Woman-only decision-making	1
Partner-only decision-making	0.85 (0.81–0.91)*
Shared decision-making	0.94 (0.90–0.99)*
**Country**	
Burkina Faso	1
Democratic Republic of Congo	0.02 (0.01–0.02)*
Côte d'Ivoire	0.22 (0.20–0.25)*
Ghana	0.17 (0.15–0.18)*
Kenya	0.60 (0.55–0.65)*
Lesotho	2.91 (2.54–3.33)*
Malawi	1.81 (1.67–1.96)*
Mali	0.24 (0.22–0.27)*
Mauritania	0.02 (0.02–0.03)*
Mozambique	0.41 (0.37–0.45)*
Nigeria	0.10 (0.09–0.11)*
Senegal	0.54 (0.49–0.58)*
Tanzania	0.30 (0.27–0.33)*
Zambia	1.65 (1.51–1.80)*
**Age group (years)**	
25–29	1
30–34	1.38 (1.30–1.46)*
35–39	1.73 (1.63–1.83)*
40–44	1.98 (1.86–2.10)*
45–49	2.07 (1.93–2.21)*
**Education**	
No education	1
Primary	1.70 (1.60–1.81)*
Secondary/higher	2.38 (2.22–2.54)*
**Wealth index**	
Poor	1 (Reference)
Middle	1.22 (1.15–1.29)*
Rich	1.69 (1.59–1.80)*

Media exposure	1.32 (1.26–1.39)*
Early marriage	1.04 (0.99–1.09)
Adolescent motherhood	1.02 (0.97–1.07)
Inequitable gender attitudes	0.94 (0.90–0.99)*
Rural residence	0.79 (0.76–0.83)*
Distance problem to health facilities	0.92 (0.88–0.96)*

^a^OR = Adjusted Odds Ratio; 95% CI = 95% Confidence Interval.

Significant cross-country differences in screening uptake were observed. Compared with Burkina Faso, women in the Democratic Republic of Congo (aOR = 0.02, 95% CI = 0.01, 0.02), Côte d'Ivoire (aOR = 0.22, 95% CI = 0.20, 0.25), Ghana (aOR = 0.17, 95% CI = 0.15, 0.18), Kenya (aOR = 0.60, 95% CI = 0.55, 0.65), Mali (aOR = 0.24, 95% CI = 0.22, 0.27), Mauritania (aOR = 0.02, 95% CI = 0.02, 0.03), Mozambique (aOR = 0.41, 95% CI = 0.37, 0.45), Nigeria (aOR = 0.10, 95% CI = 0.09, 0.11), Senegal (aOR = 0.54, 95% CI = 0.49, 0.58), and Tanzania (aOR = 0.30, 95% CI = 0.27, 0.33) had significantly lower odds of screening, whereas Lesotho (aOR = 2.91, 95% CI = 2.54, 3.33), Malawi (aOR = 1.81, 95% CI = 1.67, 1.96), and Zambia (aOR = 1.65, 95% CI = 1.51, 1.80) showed significantly higher odds of screening.

Screening uptake also increased progressively with age, with women aged 45–49 years twice as likely to report screening compared with those aged 25–29 years (aOR = 2.07, 95% CI = 1.93, 2.21). Women with secondary or higher education were more than twice as likely to undergo screening compared with those without formal education (aOR = 2.38, 95% CI = 2.22, 2.54). In addition, women from rich households (aOR = 1.69, 95% CI = 1.59, 1.80) and those exposed to mass media (aOR = 1.32, 95% CI = 1.26, 1.39) had significantly higher odds of cervical cancer screening. Conversely, women with inequitable gender attitudes (aOR = 0.94, 95% CI = 0.90, 0.99), living in rural areas (aOR = 0.79, 95% CI = 0.76, 0.83) and reporting distance to health facilities as a barrier (aOR = 0.92, 95% CI = 0.88, 0.96) were associated with lower screening uptake.

### 3.3 Country-specific variation in the association between healthcare decision-making and cervical cancer screening

There was strong evidence that the association between healthcare decision-making and cervical cancer screening uptake varied across countries (overall Wald test for interaction: χ^2^ = 96.54, df = 26, *P* < 0.01) (see supplementary Table S2). Table 3 presents the adjusted predicted probabilities of cervical cancer screening according to healthcare decision-making category across the 14 study countries. Considerable heterogeneity was observed in both the magnitude and direction of the association. For example, women who made healthcare decisions independently had the highest predicted probability of cervical cancer screening in Ghana (6.42%), Kenya (18.35%), Lesotho (48.18%), Mauritania (1.20%), Nigeria (3.73%), Tanzania (10.00%), and Zambia (35.15%). In contrast, women whose healthcare decisions were made jointly with their husband, partner, or someone else (shared decision-making) had the highest predicted probability in Burkina Faso (27.67%), Côte d'Ivoire (7.56%), Mali (7.87%), and Mozambique (13.34%), while women whose partners made healthcare decisions alone had the highest predicted probability in Malawi (36.57%) and Senegal (15.33%).

**Table 3:** Adjusted predicted probabilities of cervical cancer screening by healthcare decision-making category among ever-partnered women in 14 sub-Saharan African countries using DHS data, 2019–2024.

Country	Women alone (%)	Partner alone (%)	Shared decision-making (%)
Burkina Faso	24.39	21.51	27.67
DRC	0.98	0.51	0.57
Côte d'Ivoire	7.13	6.23	7.56
Ghana	6.42	4.37	5.14
Kenya	18.35	13.16	16.00
Lesotho	48.18	46.16	44.26
Malawi	35.05	36.57	35.11
Mali	6.11	7.12	7.87
Mauritania	1.20	0.49	0.86
Mozambique	13.10	8.07	13.34
Nigeria	3.73	3.26	3.25
Senegal	12.38	15.33	14.10
Tanzania	10.00	6.89	9.56
Zambia	35.15	33.72	32.37

Adjusted predicted probabilities were estimated from the fully adjusted logistic regression model. The interaction between healthcare decision-making and country was statistically significant (Wald χ^2^ = 96.54, df = 26, *P* < 0.001).

## 4 Discussion

Using pooled, nationally representative DHS data from fourteen SSA countries, this study examined the association between healthcare decision-making and cervical cancer screening uptake among ever-partnered women aged 25–49 years. Three key findings emerge. First, cervical cancer screening uptake across the region remains extremely low, with only 13% of women reporting ever being screened. Second, healthcare decision-making was independently associated with cervical cancer screening uptake, with women who made healthcare decision independently generally demonstrating higher screening uptake than those whose healthcare decisions were made solely by their partners or through shared decision-making. Third, the relationship between partner-only healthcare decision-making and screening uptake varied substantially across countries.

The low screening prevalence observed is consistent with previous multi-country studies and highlights the slow progress toward the WHO cervical cancer elimination targets in SSA (Bruni et al., 2022; Ba et al., 2021; Asgedom et al., 2024). Screening coverage remains particularly constrained in settings characterised by weak health systems, limited resources, and fragmented preventive services (Mwaka et al., 2016; Hull et al., 2020). The wide variation in screening prevalence across countries, ranging from approximately 1% in the Democratic Republic of Congo to over 20% in Zambia, underscores the importance of national screening policies, service availability, and implementation capacity in shaping access to preventive care (Ba et al., 2021; Hull et al., 2020).

At the pooled level, healthcare decision-making had significantly lower odds of cervical cancer screening compared with women who made healthcare decisions independently, those whose healthcare decisions were made solely by their partners or Share with thier partners or others had significantly lower odds of undergoing cervical cancer screening. This finding aligns with a growing body of literature linking women's participation in healthcare decision-making to increased utilisation of sexual and reproductive health services (Pratley, 2016; Yaya et al., 2016; Asaolu et al., 2018). Cervical cancer screening may be particularly sensitive to gender norms surrounding women's bodies, sexuality, and mobility, especially in contexts where healthcare decisions are negotiated within intimate partnerships (Petersen et al., 2022; Chol et al., 2019). Importantly, the association persisted after adjusting for education, wealth, media exposure, and other structural factors, suggesting that partner-only healthcare decision-making captures a distinct relational dimension of healthcare access beyond individual socioeconomic status. This supports frameworks that conceptualise health-seeking in relation to intra-household power relations and gendered expectations (Heise et al., 2019; Connell, 2013; Kabeer, 2016).

Consistent with prior studies, screening uptake was higher among older women, those with higher educational attainment, greater household wealth, and exposure to mass media, while rural residence and long distance to health facilities remained significant barriers (Ba et al., 2021; Baykemagn et al., 2025). These findings reinforce the persistence of socioeconomic and geographic inequities in access to preventive cancer services and suggest that progress toward elimination will require both supply-side investments and targeted demand-generation strategies. In addition, women with inequitable gender attitudes were less likely to undergo cervical cancer screening, supporting the Theory of Gender and Power, which suggests that gendered power imbalances can limit women's autonomy and access to preventive healthcare (Connell, 2013) (Baruwa, 2025), a finding consistent with a previous study in Mozambique (Baruwa, 2025).

A major contribution of this study is the demonstration that the association between healthcare decision-making and cervical cancer screening is context-dependent rather than uniform across SSA. The significant interaction between healthcare decision-making and country indicates that the relationship varies according to national sociocultural and health-system contexts. Adjusted predicted probabilities showed that women making healthcare decisions independently had the highest probability of cervical cancer screening in several countries, including Ghana, Kenya, Lesotho, Nigeria, Tanzania, and Zambia. In contrast, shared healthcare decision-making was associated with the highest predicted probability of screening in Burkina Faso, Côte d'Ivoire, Mali, and Mozambique, whereas partner-only healthcare decision-making was associated with the highest predicted probability in Malawi and Senegal. These findings suggest that no single healthcare decision-making arrangement is universally associated with higher cervical cancer screening uptake across SSA. Instead, the influence of healthcare decision-making appears to depend on broader contextual factors, including prevailing gender norms, partner involvement practices, health-system organisation, and the availability and accessibility of screening services (Asaolu et al., 2018; Ditekemena et al., 2012). Although the cross-sectional design precludes identification of the specific mechanisms underlying these differences, the findings highlight the importance of adopting context-specific approaches rather than assuming that the relationship between healthcare decision-making and preventive healthcare utilisation is consistent across countries.

These findings have important implications. Cervical cancer prevention strategies that focus exclusively on women without considering household decision-making may have limited effectiveness. Programmes should promote women's meaningful participation in healthcare decision-making while encouraging supportive partner engagement where appropriate. Evidence from maternal and HIV programs suggests that carefully designed male engagement strategies can improve service uptake when they promote shared decision-making and respect women's autonomy (Asaolu et al., 2018; Doyle et al., 2018). The observed cross-country variation highlights the importance of aligning gender-transformative interventions with national screening policies and broader health system strengthening efforts.

This study has several strengths, including the use of large, nationally representative DHS datasets, standardised measures across countries, and a comparative analytical approach that explicitly examines contextual moderation. However, limitations should be acknowledged. The cross-sectional design precludes causal inference, and the DHS measure of decision-making power may not fully capture the complexity of partner dynamics or the quality of decision-making processes. Screening uptake was self-reported and may be subject to recall or social desirability bias.

## 5 Conclusion

This study demonstrates that healthcare decision-making is a significant but context-dependent determinant of cervical cancer screening uptake among women in SSA. Although women whose healthcare decisions were made solely by their partners or shared decision-making generally had lower screening uptake in the pooled analysis, the relationship between healthcare decision-making and cervical cancer screening varied considerably across countries. These findings underscore the importance of moving beyond simplistic notions of women's autonomy by recognising that different healthcare decision-making arrangements may influence preventive healthcare utilisation differently across sociocultural and health-system contexts. Accelerating cervical cancer elimination in SSA will require gender-responsive and context-specific strategies that strengthen women's participation in healthcare decision-making while promoting supportive household and community environments alongside continued investments in health-system capacity. Future studies should explore the mechanisms underlying these country-specific differences and quantify the contribution of healthcare decision-making to inequalities in cervical cancer screening across the region.

## CRediT authorship contribution statement

**Ololade Julius Baruwa:** Writing – review & editing, Methodology, Formal analysis, Data curation, Conceptualization. **Mwiza Gideon Singini:** Writing – review & editing, Methodology, Formal analysis, Data curation, Conceptualization. **Hlengiwe Gwebu:** Writing – review & editing, Supervision, Formal analysis. **Chinelo Cynthia Nduka:** Writing – review & editing, Writing – original draft, Conceptualization.

## Consent for publication

Not applicable.

## Ethics approval and consent to participate

Not applicable.

## Funding

This study received no funding.

## Declaration of competing interest

The authors declare that they have no known competing financial interests or personal relationships that could have appeared to influence the work reported in this paper.

## Data Availability

Data will be made available on request.
