# Supplementary material for: Accelerating cervical cancer elimination: healthcare decision-making and cervical cancer screening uptake in sub-Saharan Africa
Source: Prev Med Rep. 2026 Jul 15;69:103572. doi: 10.1016/j.pmedr.2026.103572 (PMC13400434; doi:10.1016/j.pmedr.2026.103572)
Supplement: Supplementary file 1 — Supplementary material. [file mmc1.docx]

**SUPPLEMENTARY**

**Table S1: Variance inflation factor and tolerance in the multivariable regression analysis**

|  | **VIF** | **Tolerance** |
| --- | --- | --- |
| **Variables** |  |  |
| Wealth index | 1.79 | 0.558563 |
| Early marriage | 1.62 | 0.618765 |
| Adolescent motherhood | 1.57 | 0.637755 |
| Place of residence | 1.52 | 0.657323 |
| Education | 1.36 | 0.737952 |
| Media exposure | 1.18 | 0.846653 |
| Distance problem to health facilities | 1.10 | 0.909517 |
| Inequitable gender attitudes | 1.07 | 0.932157 |
| Age | 1.03 | 0.968605 |
| Country | 1.03 | 0.969647 |
| Healthcare decision-making | 1.00 | 0.996105 |
| Mean VIF | 1.30 |  |

**Supplementary Table S2. Overall test for interaction between healthcare decision-making and country**

| **Interaction term** | **Wald χ²** | **df** | **P-value** |
| --- | --- | --- | --- |
| Healthcare decision-making × Country | 96.54 | 26 | <0.001 |
